# Mouse Spermatocytes Express CYP2E1 and Respond to Acrylamide Exposure

**DOI:** 10.1371/journal.pone.0094904

**Published:** 2014-05-02

**Authors:** Belinda J. Nixon, Aimee L. Katen, Simone J. Stanger, John E. Schjenken, Brett Nixon, Shaun D. Roman

**Affiliations:** 1 Reproductive Science Group, School of Environmental and Life Sciences, University of Newcastle, Callaghan, New South Wales, Australia; 2 Australian Research Council Centre of Excellence in Biotechnology and Development, School of Environmental and Life Sciences, University of Newcastle, Callaghan, New South Wales, Australia; Northwestern University Feinberg School of Medicine, United States of America

## Abstract

Metabolism of xenobiotics by cytochrome P450s (encoded by the *CYP* genes) often leads to bio-activation, producing reactive metabolites that interfere with cellular processes and cause DNA damage. In the testes, DNA damage induced by xenobiotics has been associated with impaired spermatogenesis and adverse effects on reproductive health. We previously reported that chronic exposure to the reproductive toxicant, acrylamide, produced high levels of DNA damage in spermatocytes of Swiss mice. CYP2E1 metabolises acrylamide to glycidamide, which, unlike acrylamide, readily forms adducts with DNA. Thus, to investigate the mechanisms of acrylamide toxicity in mouse male germ cells, we examined the expression of the CYP, CYP2E1, which metabolises acrylamide. Using Q-PCR and immunohistochemistry, we establish that CYP2E1 is expressed in germ cells, in particular in spermatocytes. Additionally, *CYP2E1* gene expression was upregulated in these cells following *in vitro* acrylamide exposure (1 µM, 18 h). Spermatocytes were isolated and treated with 1 µM acrylamide or 0.5 µM glycidamide for 18 hours and the presence of DNA-adducts was investigated using the comet assay, modified to detect DNA-adducts. Both compounds produced significant levels of DNA damage in spermatocytes, with a greater response observed following glycidamide exposure. A modified comet assay indicated that direct adduction of DNA by glycidamide was a major source of DNA damage. Oxidative stress played a small role in eliciting this damage, as a relatively modest effect was found in a comet assay modified to detect oxidative adducts following glycidamide exposure, and glutathione levels remained unchanged following treatment with either compound. Our results indicate that the male germ line has the capacity to respond to xenobiotic exposure by inducing detoxifying enzymes, and the DNA damage elicited by acrylamide in male germ cells is likely due to the formation of glycidamide adducts.

## Introduction

Paternal exposure to environmental toxicants or xenobiotics has been associated with adverse reproductive effects such as birth defects, miscarriages, and childhood genetic diseases [1]. The potential for xenobiotics to induce genetic damage in male germ cells is thought to be involved in mediating these reproductive effects, as the male germ line has limited capacity for DNA repair, particularly in the later stages of spermatogenesis [2], [3], [4]. The genotoxic impact of xenobiotics may also be amplified (bio-activated) following their metabolism via detoxifying enzymes, such as the CYPs. CYP2E1 is one of several CYPs known to cause bio-activation and metabolises a range of exogenous substances, including acrylamide [5], [6]. Acrylamide is of particular interest, as traces of the compound have been detected in numerous carbohydrate-rich foods such as potato chips and breads [7], [8]. Additionally, acrylamide is a known neurotoxicant in humans and acts as a carcinogen, genotoxin and reproductive toxin in rodents [9]. Whilst the reproductive toxicity of acrylamide has not been observed in humans to date, there are concerns that chronic dietary exposure to the compound may have a cumulative effect on human fertility and reproductive health [10].

Interestingly, the reproductive toxicity of acrylamide primarily affects the male. Acrylamide is readily distributed throughout the body, is able to transit the blood testis barrier in rodents and has been shown to accumulate in the testis within one hour of oral exposure [11]. Rodent studies have reported decreases in copulatory behaviour and a loss of spermatogenesis following exposure to acrylamide in males [12]. Multi-generational effects have also been described, such as the loss of post implantation embryos and reduced postnatal survival. In a study by Sakamoto and Hashimoto [13], high doses of acrylamide in the drinking water of male mice lead to decreases in fertility, reduced litter sizes, and increases in embryo resorptions. However, the mechanisms by which acrylamide elicits these reproductive effects are not clear. Based on the timing between exposure and effect (loss of embryos), sperm transit of the epididymis has been the major focus, with failure to fertilise attributed to protamine alkylation in maturing sperm [14], [15]. Alternatively, a clastogenic mechanism may be involved, as the formation of kinesin adducts in the meiotic/mitotic spindles may impair chromosomal segregation in germ cells during spermatogenesis [16].

The above mechanisms require the interaction of acrylamide with an intermediary protein, as acrylamide does not directly react with DNA and preferentially forms adducts with cysteine residues of proteins [17]. However, the metabolism of acrylamide by CYP2E1 generates the epoxide metabolite, glycidamide, which has a higher mutagenic potential than acrylamide and directly interacts with DNA, forming adducts [18]. Indeed, the presence of these adducts have been identified in the lung, liver, kidney and testes following acrylamide exposure in mice [19]. Studies by Ghanayem *et al*. [20], [21], [22] have also demonstrated the significance of the metabolic conversion of acrylamide to glycidamide in CYP2E1 knockout mice. The multigenerational effects were abrogated in CYP2E1-null males, indicating that the presence of CYP2E1 is essential in mediating acrylamide reproductive toxicity. It is therefore hypothesised that the reproductive effects of acrylamide are related to its conversion to glycidamide, which generates DNA adducts, leading to genetic damage in the male germ line.

We recently reported that chronic exposure of male mice to acrylamide at doses relevant to human exposure lead to significantly increased levels of DNA lesions in spermatocytes [23]. Given that acrylamide is metabolised by CYP2E1, the expression and regulation of this CYP was examined in the present study within specific stages of early male germ cell development. We show that spermatocytes express CYP2E1, indicating that they have the capacity to metabolise acrylamide to glycidamide. Furthermore, the DNA damage induced in spermatocytes by acrylamide or its metabolite, glycidamide, following *in vitro* exposure was examined using the comet assay, modified to detect DNA adducts. The results of the present study support the notion that DNA damage induced in meiotic germ cells by acrylamide is likely attributable to the formation of DNA adducts generated by glycidamide.

## Materials and Methods

### Animal Ethics Statement

Experiments involving animals were conducted in strict accordance with the policies set out by the Animal Care and Ethics Committee of the University of Newcastle (Ethics Numbers: SR1004 0708, A-2008-145). Swiss mice were housed under conditions of 16 hours light, 8 hours dark, with food and water provided *ad libitum*. Animals were euthanized by CO_2_ asphyxiation and all efforts were made to minimize suffering.

### Chemicals and reagents

All chemicals and reagents including custom designed primers were obtained from Sigma Chemicals (St Louis, MO) unless otherwise stated, and were of molecular biology or research grade. Rabbit polyclonal anti-cytochrome p450 2E1 antibody (anti-CYP2E1, ab28146) was obtained from Abcam (Cambridge, MA). Mouse anti-cAMP dependent Protein Kinase [Catalytic subunit] antibody (anti-PKA[C], #610981) was purchased from BD Transduction Laboratories. Rat anti-germ cell nuclear antigen antibody (anti-GCNA) was a gift from Dr. George Enders [24]. Secondary antibodies, Alexa Fluor 594 goat anti-rabbit immunoglobulin G (IgG) (A11012) was purchased from Invitrogen (Carlsbad, CA). Dulbecco's Modified Eagle Media (DMEM) and supplements for cell culture were obtained from Sigma and Invitrogen. Acrylamide was obtained from Sigma (≥99% purity, A9099) and glycidamide (98% purity, G615250) was obtained from Toronto Research Chemicals (North York, CA). Oligo(dT)15 primer, RNasin, dNTPs, M-MLV-Reverse Transcriptase, RQ1 DNase, GoTaq Flexi, MgCl_2_ and GoTaq quantitative PCR master mix were obtained from Promega (Madison, WI). DNA repair endonucleases, formamidopyrimidine-DNA glycosylase (FPG) and 8-oxoguanine DNA glycosylase (hOGG1) were purchased from New England Biolabs Inc. (Arundel, Qld).

### Germ cell isolation

Spermatogonia, spermatocytes and spermatids (Fig S1 in File S1) were enriched from dissected mouse testes using density sedimentation at unit gravity as described previously [25]. Briefly, testes were disassociated and tubules were digested sequentially with 0.5 mg/ml collegenase/DMEM and 0.5% v/v trypsin/EDTA to remove extra-tubular contents and interstitial cells. Remaining cells were loaded onto a 2–4% w/v bovine serum albumin (BSA)/DMEM gradient to separate male germ cell types according to density. Germ cell fractions were collected, washed and counted. This method enables the isolation of germ cells with very little to no somatic cell contamination, as extra-tubular cells are digested and removed prior to density sedimentation. Greater than 90% purity can be achieved for spermatogonial isolations. Spermatocye isolation achieves 65–70% purity for pachytene spermatocyte. The remaining cells in these isolations consist of early leptotene, zygotene and diplotene spermatocytes. For isolation of round spermatids the purity is 85–95% with contaminating cells consisting of late spermatids [25].

### RNA extraction

Total RNA was isolated from male germ cells and whole testis using two rounds of a modified acid guanidinium thiocyanate-phenol chloroform protocol [26], in which cells and tissues were lysed with lysis buffer (4 M guanidinium thiocyanate, 25 mM sodium citrate, 0.5% v/v sarkosyl, 0.72% v/v β-mercaptoethanol). RNA was isolated by phenol/chloroform extraction and isopropanol precipitated.

### Quantitative PCR (Q-PCR)

Reverse transcription was performed with 2 µg of isolated RNA, 500 ng oligo(dT)15 primer, 40 U of RNasin, 0.5 mM dNTPs, and 20 U of M-MLV-Reverse Transcriptase. Total RNA was DNase treated prior to reverse transcription to remove genomic DNA contamination. Q-PCR was performed using SYBR Green GoTaq qPCR master mix according to manufacturer's instructions on an MJ Opticon 2 (MJ Research, Reno, NV, USA). The sequences of all primers used in this study and the predicted size of the amplicons are provided in Table S1 in File S1. Reactions were performed on cDNA equivalent to 100 ng of total RNA and carried out for 40 amplification cycles. SYBR Green fluorescence was measured after the extension step at the end of each amplification cycle and quantified using Opticon Monitor Analysis software Version 2.02 (MJ Research). Each sample was examined in triplicate and a replicate omitting the reverse transcription step was undertaken as a negative control. Real-time data were normalized to cyclophilin expression using the equation 2e-ΔC(t), where C(t) is the cycle at which fluorescence was first detected above background fluorescence [27]. Real-time data were presented as the average of each replicate, normalized to each reference sample (±SEM). Further validation of Q-PCR data was conducted using the geometric mean of cyclophilin and a second reference gene, hypoxanthine-guanine phosphoribosyltransferase (hprt),in accordance with Vandesompele et al. [28] (Fig. S2 in File S1). The 2-ΔΔC(t) transformation [27] was used for comparisons between treated and vehicle control primary cultures. Values for each replicate were averaged, and relative expression levels between treated samples were depicted as percentages of controls (±SEM). Each data set is the average of at least three separate experiments.

### Immunohistochemistry & immunocytochemistry

Mouse testes were fixed in Bouin's fixative, embedded in paraffin wax and sectioned at 5 µm thickness. Sections were de-paraffinized, rehydrated, and antigen retrieval was performed using Proteinase K (20 µg/ml) for 30 min at room temperature. Sections were blocked in 3% w/v BSA/phosphate buffered saline with 0.05% v/v Tween-20 (PBST) for 1 h at room temperature, after which they were incubated with anti-CYP2E1 (1∶50 with 1% w/v BSA/PBST) overnight at 4°C. Sections were then washed and incubated with fluorescent-conjugated secondary antibody, Alexa Fluor 594 goat anti-rabbit IgG (1∶200 with 1% w/v BSA/PBST), for 1 h at room temperature. Counterstaining was conducted using '-6-diamidino-2-phenylindole (DAPI) for 3 min. Sections were then mounted in Mowiol and observed under fluorescence on an Axio Imager A1 fluorescent microscope (Carl Zeiss MicroImaging, Inc., Thornwood, NY). As a control, parallel testis tissue sections were probed with rabbit serum in the absence of primary antibody, which did not produce a detectable signal (blank images not shown). Images were taken using an Olympus DP70 microscope camera (Olympus America, Centre Valley, PA).

Isolated spermatogonia, spermatocytes and spermatids were air dried onto a 12 well slide and blocked in 3% w/v BSA/PBST for 1 h at room temperature. Germ cells were dual-stained with anti-CYP2E1 (1∶50 with 1% w/v BSA/PBST) and anti-GCNA (1∶20 with 1% w/v BSA/PBST) or anti-PKA[C] (1∶25 with 1% w/v BSA/PBST) for 1 h at room temperature. Anti-GCNA labels spermatogonia and spermatocytes whereas anti-PKA[C] is a marker for spermatids [24], [29]. Cells were then washed and incubated with appropriate secondary antibodies for 1 h at room temperature. As a control, cells were also probed with rabbit serum in the absence of primary antibody, which did not produce a detectable signal (blank images not shown), and slides were subsequently viewed under fluorescence as previously described above.

### Treatment of germ cells with chemicals

Isolated germ cells were suspended in 1 ml sterile DMEM, supplemented with 100 µM sodium pyruvate, 200 µM L-glutamate, 100 U/ml penicillin, 10 µg/ml streptomycin, and 5% v/v fetal bovine serum. Cells were treated with acrylamide at a final concentration of 10 nM, 100 nM, 1 µM or 10 µM or glycidamide at a final concentration of 5 nM, 50 nM, 0.5 µM or 50 µM and all samples were incubated for 18 h at 37°C, 5% CO_2_. In a previous study, treatment of dissociated testicular cells with acrylamide or glycidamide was carried out at millimolar concentrations, for relatively short exposure times of 2 h (Hansen et al. 2010). Therefore, due to the length of exposure used in the current study (18 h), the concentration of acrylamide was adjusted to the 10 nM–10 µM range to ensure cell viability remained largely unaffected whilst still eliciting a cellular response to chemical exposure in spermatocytes. Glycidamide was found to elucidate similar effects to acrylamide at lower doses of 5 nM–5 µM. Negative control samples were treated with vehicle only (distilled water, dH_2_O). An additional control in which germ cells were treated at room temperature with hydrogen peroxide (H_2_O_2_) at a final concentration of 500 µM for 5 min was also included. In some experiment cells were treated with resveratrol at a final concentration of 0.1 µM (suspended in ethanol at a final concentration of 1%) in the presence or absence of acrylamide (1 µM) or glycidamide (0.5 µM) for 18 hours as described. In this instance the negative control samples were treated with 1% ethanol. Following treatment, cell suspensions were washed and collected by centrifugation prior to further analysis.

### Trypan Blue exclusion and germ cell staining

Germ cells exposed to acrylamide were assessed for cell viability using trypan blue live/dead staining. Cells were stained with 0.08% v/v trypan blue and more than 200 cells per replicate were scored using a haemocytometer. To observe germ cell morphology following acrylamide treatment, cells were fixed in 4% v/v paraformaldehyde and 1×10^4^ cells were air dried onto a 12-well slide. Cells were permeabilised with 0.2% v/v Triton X-100/PBS for 10 min at room temperature and then incubated with peanut lectin conjugated to fluorescein isothiocyanate (FITC-PNA) for 15 min at room temperature. FITC-PNA fluorescently labels the developing acrosome green and can be used to distinguish different spermatogenic cell types. Cells were counterstained with propidium iodide (PI), mounted in Mowiol, and visualised using fluorescence microscopy as previously described above.

### Determination of DNA damage in germ cells using the comet assay

The extent of germ cell DNA damage elicited by exposure to acrylamide was assessed using an alkaline comet assay according to methods published by [30] with modifications detailed below. Fully frosted Dakin slides (ProSciTech, Australia) were coated with one layer of 1% w/v normal melting point agarose. Ten µl of germ cells suspended in PBS (1×10^7^ cells/ml) were mixed with 70 µl of 0.5% w/v low-melting point agarose, and this suspension was layered onto the pre-coated slides and covered with a coverslip. Once agarose was set, the coverslip was removed and cells were immersed in fresh lysis solution (2.5 M NaCl, 100 mM Na_2_EDTA, 10 mM Trizma and 1% v/v Triton X-100 at pH 10) for 1 h at 4°C. Cells were incubated in lysis buffer with dithiothreitol (10 mM final concentration) for a further 30 min at 4°C, after which lithium diiododsalicyclate was added (4 mM final concentration) and cells were incubated for 1.5 h at room temperature. Following lysis, cells were treated with adduct specific cleavage enzymes, formamidopyrimidine glycosylase (FPG) or 8-oxoguanine DNA glycosylase (hOGG1) at 1∶1000 [0.4 units/gel] and 1∶500 [0.16 units/gel] respectively, for 30 min at 37°C (enzyme optimisation data included in Fig. S3 in File S1). Control cells were treated with enzyme buffer (40 mM HEPES, 0.1 M KCl, 0.5 mM EDTA, 0.2 mg/ml BSA, pH 8.0 with KOH) and all samples were incubated in chilled alkaline electrophoresis buffer (0.3 M NaOH, 1 mM EDTA) for 20 min. Electrophoresis was carried out for 5 min at 0.9 V/cm, 300 mA, after which slides were drained and neutralised (0.4 M Tris, pH 7.5). Slides were stained with SYBR green (Trevigen, Gaithersburg, MD) and visualised under fluorescence. The DNA integrity of 50–100 cells per slide was analysed using Comet Assay IV software (Perceptive Instruments, Suffolk, UK). The fluorescence intensity in the comet “tail” was used as measure of DNA damage (Tail DNA %). Highly damaged cells with irregular or blown out nuclei, referred to as ‘clouds’ or ‘hedgehogs’, were excluded from analysis, and no statistically significant differences in the frequency of ‘hedgehog’ comets was found across treatments (Fig S4–S5 in File S1).

### Quantification of cellular glutathione

Levels of glutathione (GSH) in germ cells and P19 embryonal carcinoma cells were quantified using GSH-Glo™ Glutathione Assay kit (Promega) according to manufacturers' instructions. Luminescence was analysed using a luminometer plate reader (FLUOstar Optima, BMG Labtech) and data presented is the average of three replicate experiments.

### Statistics

Statistical analyses were performed using JMP software Version 9 (SAS Institute, Cary, NC). Data was tested for normality using the Shapiro-Wilk test. When data was not from the Gaussian distribution, the non-parametrical Kruskal-Wallis test was applied. If a statistically significant difference was found across groups of means, then a post-hoc Steel-Dwass multiple comparisons test was used to examine significant differences between pairs of groups. Differences between control and treated samples were considered to be statistically significant if the probability of the difference being due to chance was less than 5% (*p*<0.05) and *F* statistic and degrees of freedom are indicated in parentheses in Figure captions. All experiments were replicated at least three times with independent samples and data are presented as the mean values ± SEM.

## Results

### Expression of CYP2E1 in the male germ line

**Figure 1:**
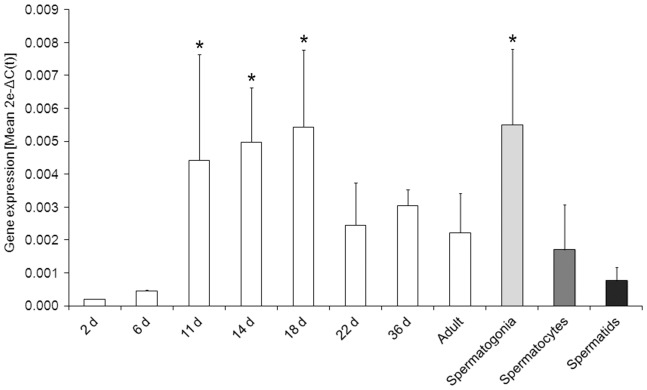
Gene expression of CYP2E1 in the male germ line. Q-PCR analysis of CYP2E1 mRNA expression in mouse testis at different developmental stages 2, 6, 11, 14, 18, 22, and 36 d after birth, and adult (older than 56 d). Expression was also examined in isolated male germ cells, spermatogonia, spermatocytes and spermatids. Data are representative of n = 3 experiments and depicted as transformed values, 2e−ΔC(t) (Mean ±SEM), as described in Materials and Methods. CYP2E1 gene expression was found predominantly in spermatogonia. Statistically significant differences were found in spermatogonia, 11, 14 and 18 d compared to 2 d testis (*F*_10,79_ = 4.0,*p<0.05).

Characterisation of *CYP2E1*gene expression within the male germline was performed by assessing the levels of expression in whole mouse testis of mice at different ages; from 2 d after birth to adult (older than 56 d) (Fig. 1). Immature testis from 2 to 6 d exhibited limited expression of*CYP2E1*. However, *CYP2E1*gene expression was maintained at relatively high levels between 11 to 18 d after birth, after which the level of *CYP2E1*was approximately halved and remained at this level through to adulthood. Examination of RNA from isolated germ cells by Q-PCR indicated that the highest levels of *CYP2E1*expression were in spermatogonia, compared to spermatocytes and spermatids. Spermatogonia are present in the testis after the migration of gonocytes to the basal membrane (4–6 d after birth) [31]. Thus, the high testicular expression of *CYP2E1*from day 11 to 18 (Fig. 1) is likely attributed to the relatively high proportion of spermatogonia that dominate the testicular environment during this early stage of development. In contrast, later stage germ cells including round and elongating spermatids, begin to develop in mouse testis at day 20 [29], and since neither of these cell types express high levels of *Cyp2e1*, the overall *Cyp2e1* expression level diminishes in maturing testis.

**Figure 2:**
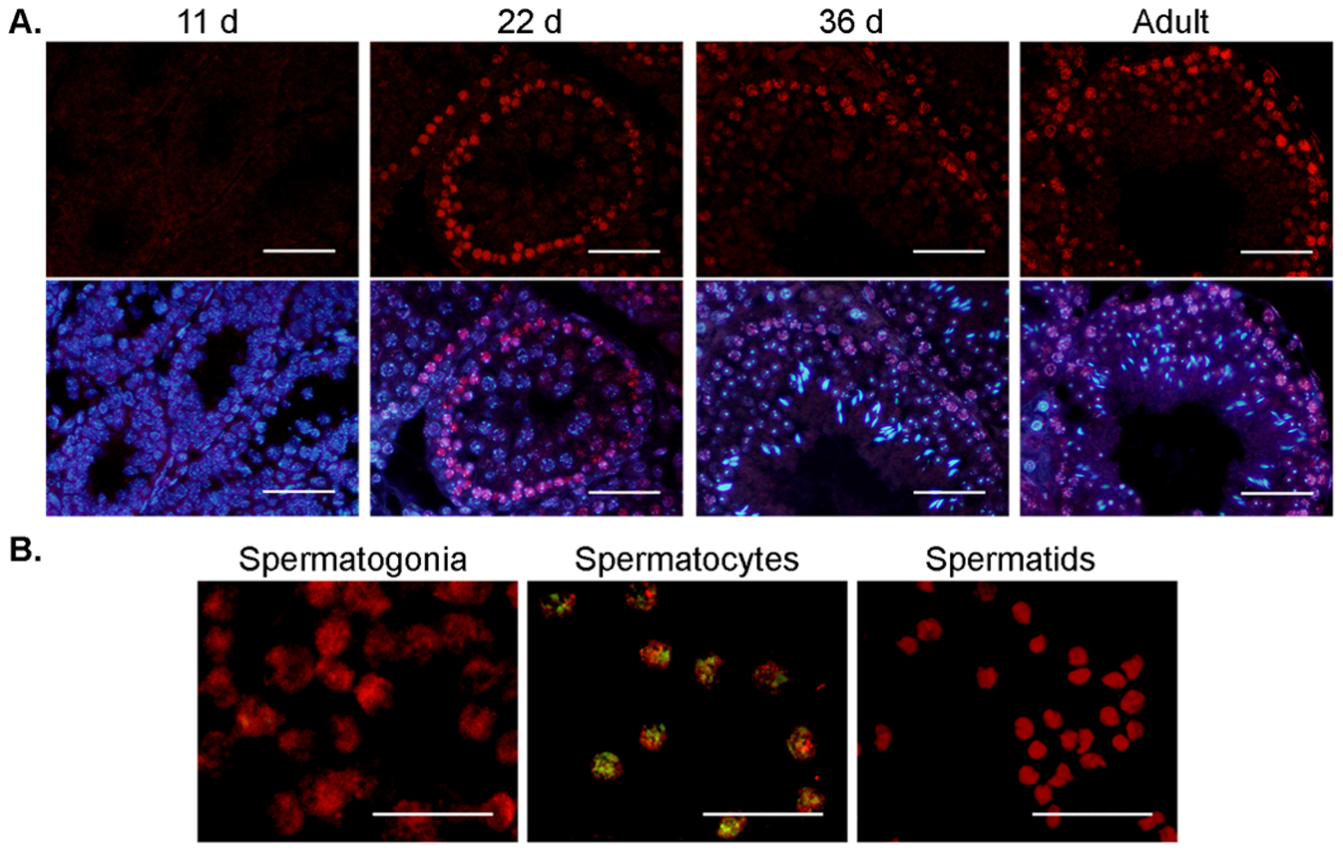
Protein expression of CYP2E1 in the male germ line. (A) Immunolocalisation of CYP2E1 (red staining) in testis sections at different developmental stages, 11 d after birth, 22 d after birth, 36 d after birth, and adult (older than 56 d). Sections were sequentially probed with anti-CYP2E1 and appropriate secondary antibody before being counter-stained with DAPI (blue staining). CYP2E1 protein expression was found in spermatocytes at 22 d after birth to adult testis, with weaker staining observed in spermatids. Diagram outlining where different male germ cell types reside in the seminiferous tubule is shown in supplementary data. (B) Immunolocalisation of CYP2E1 in isolated spermatogonia, spermatocytes and spermatids, showing CYP2E1 expression (green staining) in spermatocytes. Germ cells were probed with anti-CYP2E1 and anti-GCNA, which labels spermatogonia and spermatocytes, or anti-PKA[C], which labels spermatids (red staining). Both tissue sections and cells were probed with rabbit serum in the absence of primary antibody as a control, which did not produce a detectable signal (blank images not shown). Scale bars equal to 50 µm.

Interestingly, *Cyp2e1* appeared to be subject to translational repression. Immunohistochemistry and immunocytochemistry on isolated cells revealed that the CYP2E1 protein was predominantly expressed in the early pachytene spermatocyte stage of spermatogenesis, which are present in the testis from 14 d after birth (Fig. 2 A and B). Downregulation of CYP2E1 protein expression was observed in spermiogenic stages in 36 d old and adult testis (a diagram of germ cell stages of spermatogenesis in the testis is included in Fig. S1 in File S1). Such differences in gene and protein expression are likely the result of translational delay and repression, commonly found during the process of spermatogenesis [32]. The presence of CYP2E1 protein within pachytene spermatocytes suggests that the metabolism of acrylamide to glycidamide is likely to occur in this germ cell type. It is already established that acrylamide can accumulate in the testis and penetrate the blood testis barrier to reach these germ cells [11]. Thus, as translation of CYP2E1 mRNA appeared to be delayed in spermtogonia and spermatids exhibited relatively little CYP2E1 protein expression, investigation into the effects of acrylamide exposure was primarily focussed on spermatocytes in the present study.

### Male germ cells respond to acrylamide by increasing CYP gene expression

**Figure 3:**
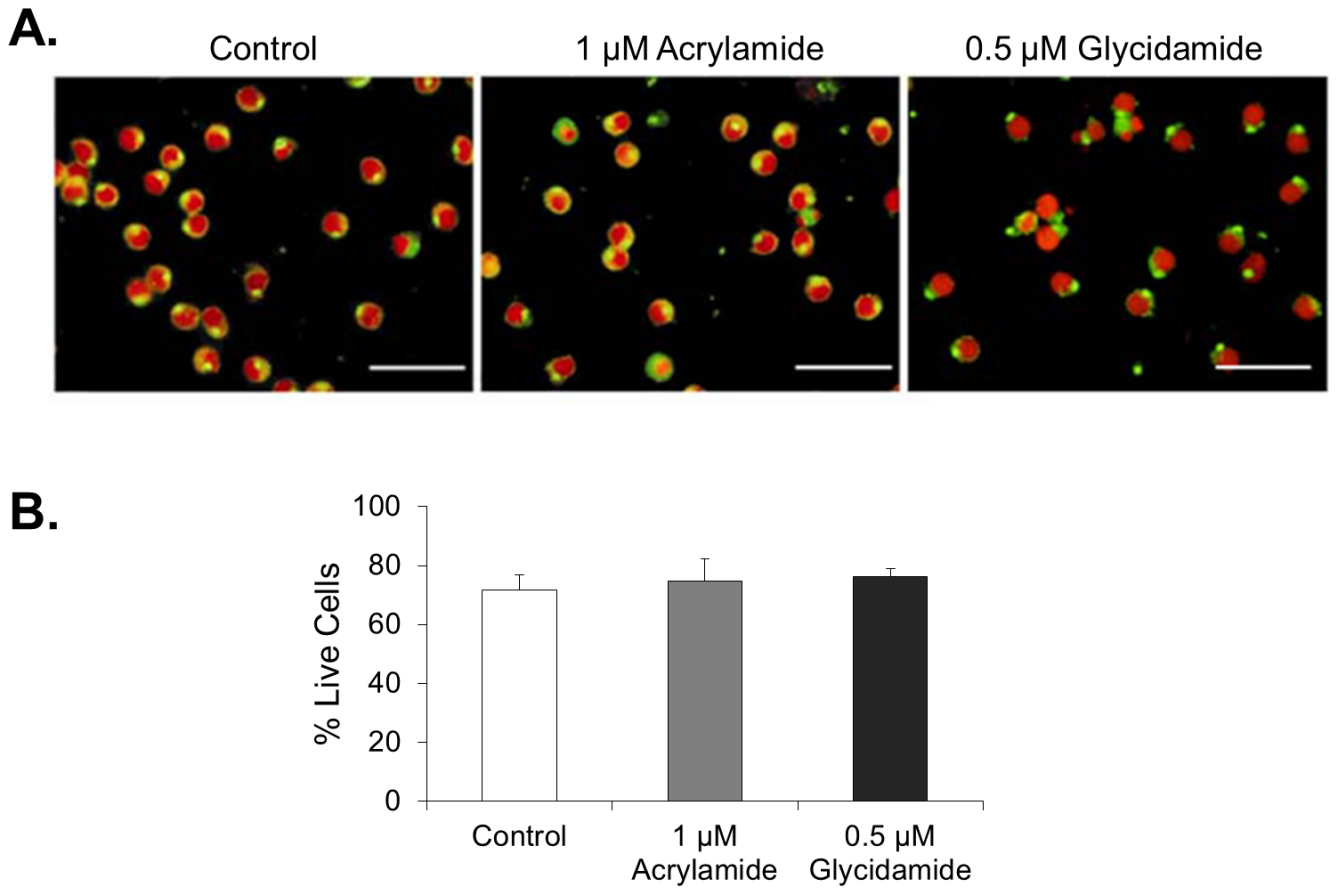
Acrylamide treatment (1 µM, 18 h) or glycidamide treatment (0.5 µM, 18 h) did not impact on spermatocyte morphology or viability. (A) Adult mouse spermatocytes were treated with acrylamide (1 µM, 18 h) or glycidamide (0.5 µM, 18 h) and dual stained with FITC-PNA, which labels the developing acrosome (green), and PI (red) to observe cell morphology. Scale bar is equal to 50 µm. (B) The viability of spermatocytes treated with acrylamide (1 µM, 18 h) or glycidamide (0.5 µM, 18 h) assessed by trypan blue exclusion. Data are representative of n = 3 experiments, measured in triplicate (Mean, ±SEM), and >200 cells were scored per replicate. At the doses used in the current study, no differences in cell morphology or viability were observed following treatment with acrylamide or glycidamide.

**Figure 4:**
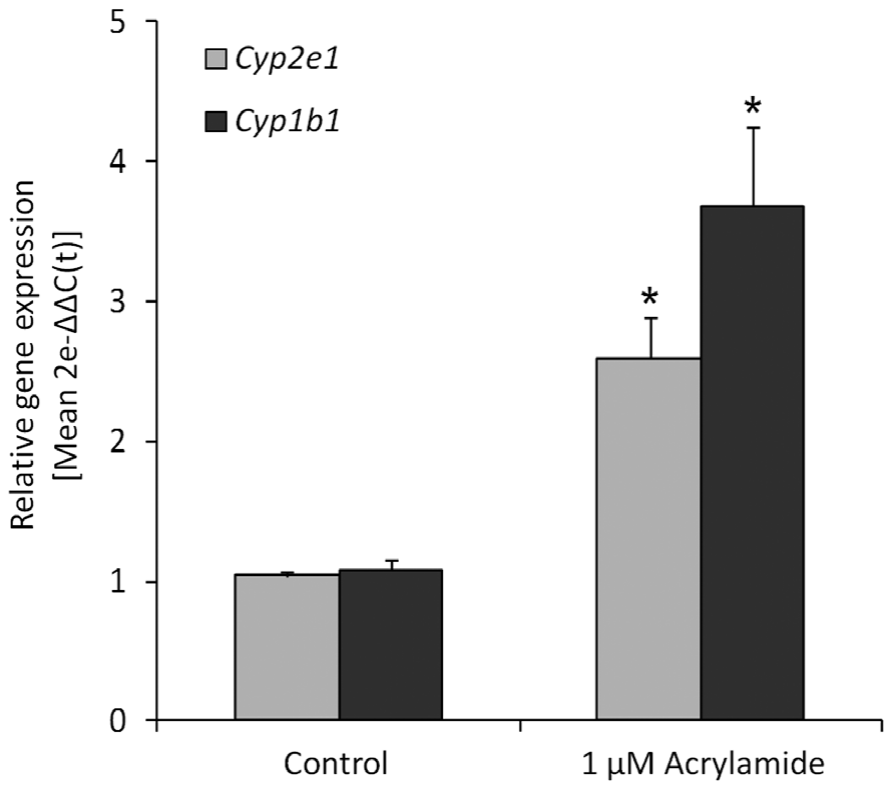
Acrylamide exposure elicits an increase in CYP gene expression in the male germ line. Gene expression levels were analysed in isolated spermatocytes by Q-PCR following incubation with acrylamide. Cells were isolated and cultured with acrylamide (1 uM, 18 h), RNA was extracted, reverse transcription performed and QPCR conducted as described in Materials and Methods. CYP2E1 and CYP1B1 gene expression was significantly increased in spermatocytes following acrylamide exposure (*F*_1,16_ = 20.1, *p<0.05). Data are depicted as transformed values (2e−ΔΔC(t)) as described in Materials and Methods, and is representative of n = 3 experiments (Mean ±SEM).

One feature of cells responding to xenobiotic exposure is the induction of xenobiotic metabolising enzymes. Indeed, upregulation of numerous P450s in response to xenobiotic exposure has previously been observed in liver tissue of mice [[33] *CYP* genes, *CYP2E1* and *CYP1B1*, were therefore examined in isolated spermatocytes following acrylamide exposure by Q-PCR, as preliminary data indicated that these genes were both constitutively expressed in these cells (data not shown). As previously mentioned, CYP2E1 specifically metabolises acrylamide; CYP1B1 however, is a known detoxifying CYP that facilitates the metabolism of polycyclic hydrocarbons as well as endogenous compounds such as estradiol and retinoic acids [6], [34], [35]. As described in the Materials and Methods section, treatment of germ cells with acrylamide was carried out at 1 µM for 18 h as this level of exposure did not affect cell viability and morphology, which was confirmed by fluorescent cell staining and trypan blue exclusion (Figs. 3 A and B). Q-PCR analysis indicated that pachytene spermatocytes responded to acrylamide exposure by significantly increasing CYP2E1 gene expression by approximately 2.5 fold compared to controls (*p*<0.05, Fig. 4). Whilst CYP1B1 does not play a role in acrylamide metabolism, it was interesting to note that acrylamide exposure also led to a significant upregulation of *CYP1B1*expression in spermatocytes.

### *In vitro* exposure to acrylamide or glycidamide induces DNA adducts in male germ cells

**Figure 5:**
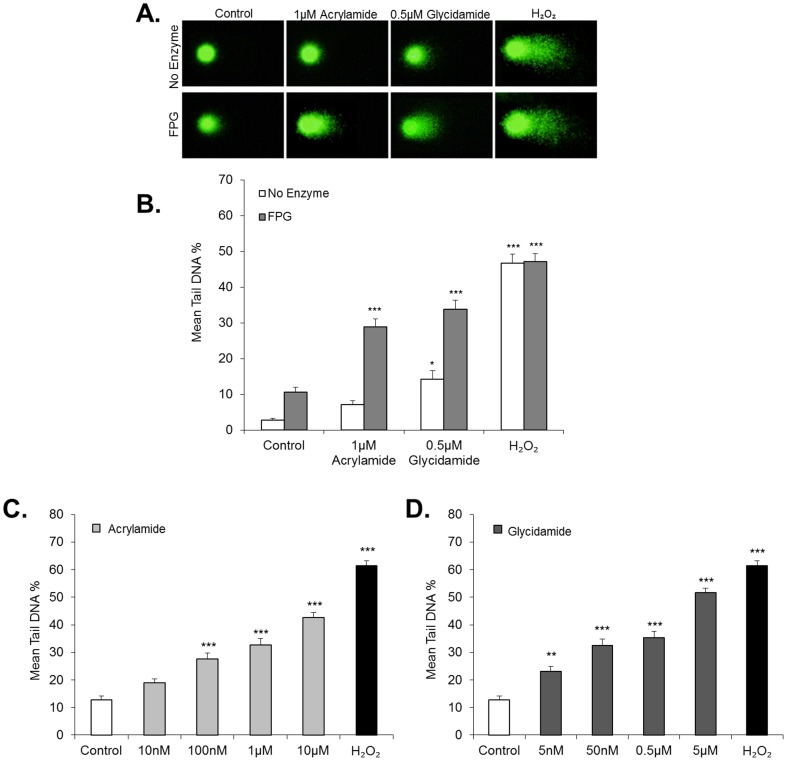
Acrylamide and glycidamide induces DNA damage in the male germ line. DNA damage was assessed in spermatocytes treated with acrylamide or glycidamide using the comet assay in the presence or absence of FPG. (A) Representative comet images from control, acrylamide (1 µM, 18 h), glycidamide (0.5 µM, 18 h) and H_2_O_2_ (500 µM, 5 min) treated spermatocytes. (B) The average Tail DNA % was assessed for each sample and in the absence of FPG, a modest increase in Tail DNA % was observed in spermatocytes treated with glycidamide (*F*_7,663_ = 61.7, *p<0.05). In the presence of FPG however, both acrylamide and glycidamide produced significant increases in Tail DNA % (***p<0.001) with a greater response observed following glycidamide exposure. Treatment of spermatocytes with H_2_O_2_ (500 µM, 5 min) was used as a positive control for damage, and induced significant increases in Tail DNA % in both the presence and absence of FPG. (C) Spermatocytes were assessed for DNA damage using the FPG comet assay following acrylamide exposure, at doses between 10 nM to 10 µM for 18 h. Spermatocytes treated with H_2_O_2_ (500 µM, 5 min) were used as a positive control for DNA damage. Significant increases in Tail DNA % were observed in spermatocytes following 100 nM acrylamide treatment and above (*F*_5,507_ = 83.6, ***p<0.001). Significant increases in Tail DNA % were also observed in cells treated with H_2_O_2_ (***p<0.001).(D) Spermatocytes were assessed for DNA damage using the FPG comet assay following glycidamide exposure, at doses between 5 nM to 5 µM for 18 h (*F*_5,491_ =  **p<0.01). Spermatocytes treated with H_2_O_2_ (500 µM, 5 min) were used as a positive control for DNA damage. Significant increases in Tail DNA % were observed in spermatocytes following 5 nM glycidamide treatment and above. All data are representative of n = 3 experiments (Mean ±SEM).

The expression of CYP2E1 in spermatocytes suggests that acrylamide is metabolised to glycidamide in these cells and may lead to the generation of DNA adducts. Thus, the DNA integrity of spermatocytes was examined using the alkaline comet assay, following either acrylamide at 1 µM or glycidamide at 0.5 µM for 18 h. A significant increase in DNA damage (Tail DNA %) was observed following glycidamide exposure (*p*<0.05); however, only a modest effect was found in cells exposed to acrylamide (Fig. 5 B). The sensitivity of the comet assay was therefore enhanced by utilising the endonuclease FPG, which recognises and introduces strand breaks at sites of DNA adducts. The inclusion of FPG led to greater detection of DNA damage in both acrylamide and glycidamide treated cells (*p*<0.001), with slightly higher levels of damage observed in the glycidamide treated sample (Fig. 5 B).

Acrylamide and glycidamide were also found to elicit a dose-dependent increase in DNA damage in the comet assay, in the presence of FPG (Fig. 5 C and D). Statistically significant increases were observed at doses of 100 nM, 1 µM and 10 µM of acrylamide after 18 hours of exposure in spermatocytes (*p*<0.001). Spermatocytes appeared to be more sensitive to glycidamide treatment, with significant increases in damage observed at exposures as low as 10 nM (*p*<0.01). H_2_O_2_ treatment (500 µM, 5 min) was used as a positive control in these experiments and generated significant increases in damage in both the presence and absence of FPG (Fig. 5 B, C and D).

### Oxidative adducts contribute little to the DNA damage induced by acrylamide in male germ cells

Exposure to acrylamide or glycidamide may lead to the generation of oxidative adducts as both compounds can be conjugated with GSH, which plays a critical role in anti-oxidant defence. Indeed, the FPG endonuclease recognises a range of DNA lesions, including oxidative adducts; thus the DNA damage induced by acrylamide or glycidamide in spermatocytes was further characterised using an alternate cleavage enzyme, hOGG1, in the comet assay. The hOGG1 enzyme specifically recognises oxidative DNA adducts, such as 8-oxo-7,8-dihydroguanine (8-oxoGua) which is an adduct induced by reactive oxygen species [36]. The use of hOGG1 in the comet assay did not produce a significant increase in Tail DNA % in spermatocytes treated with acrylamide (1 µM, 18 h); although a modest increase was observed following treatment with glycidamide. These results suggest that oxidative adducts represent a fraction of the damage induced by the acrylamide metabolite, glycidamide; in spermatocytes however, acrylamide does not directly generate oxidative DNA damage in these cells.

**Figure 6:**
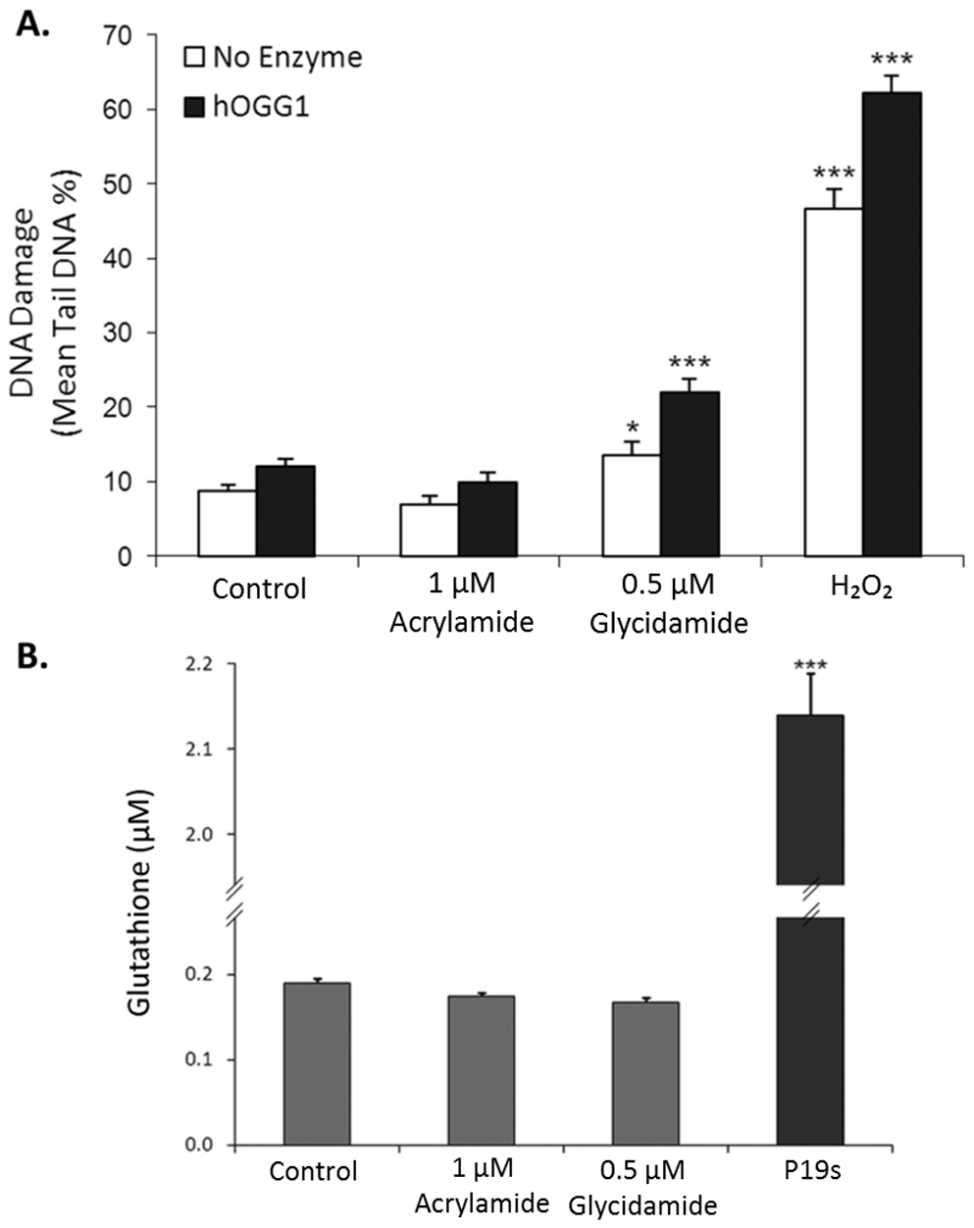
Oxidative stress may play a role in the DNA damage induced by acrylamide in spermatocytes. (A) The comet assay was conducted on spermatocytes treated with acrylamide (1 µM, 18 h) or glycidamide (0.5 µM, 18 h) in the presence or absence of hOGG1. Significant levels of DNA damage were not detected in the acrylamide treated spermatocytes in either the presence or absence of hOGG1. A modest but significant increase in Tail DNA % was observed in spermatocytes following glycidamide treatment in the absence of hOGG1 (*F*_7,677_ = 134.4, *p<0.001); however, hOGG1 treatment resulted in greater detection of Tail DNA % following glycidamide exposure. Treatment with H_2_O_2_ (500 µM, 5 min) was used as a positive control and produced significant increases in Tail DNA % in the presence of either enzyme (***p<0.001). (B) Glutathione (GSH) levels in spermatocytes were measured using a GSH assay following acrylamide (1 µM, 18 h) or glycidamide exposure (0.5 µM, 18 h). No significant differences in GSH levels in male germ cells were observed following acrylamide or glycidamide treatment. Additionally, relatively low GSH levels were found in spermatocytes (0.19 µM) compared to P19 embryonal carcinoma cells (2.14 µM, *F*_3,6_ = 1676.2, ***p<0.001). Data are representative of n = 3 experiments (Average ±SEM).

Since both acrylamide and its metabolite, glycidamide, can be conjugated with glutathione (GSH) GSH levels were measured in spermatocytes treated with acrylamide using an established GSH assay (Fig. 6 B). Intriguingly, spermatocytes have relatively low basal levels of cellular GSH compared to that of a control cell line (P19 embryonal carcinoma cells). No significant differences in GSH levels were observed after exposure of spermatocytes to acrylamide or glycidamide. These data indicated that antioxidant levels are constitutively low in spermatocytes and that oxidative damage is unlikely to be a major consequence of acrylamide or glycidamide exposure in these cells.

### Acrylamide is metabolized in spermatocytes to glycidamide via CYP2E1

**Figure 7:**
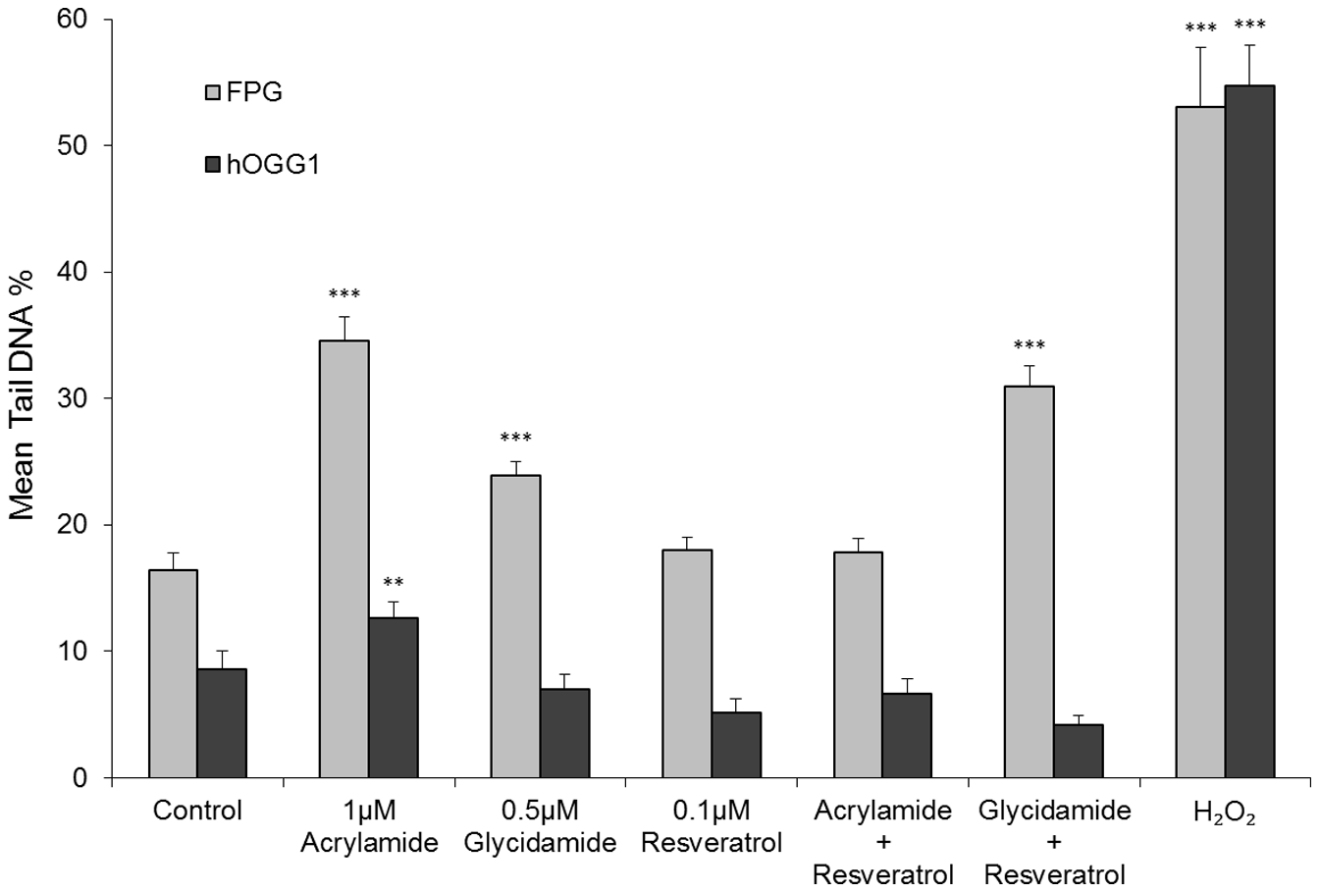
Acrylamide is metabolized in spermatocytes to glycidamide via CYP2E1. The Isolated spermatocytes were treated with acrylamide (1 µM, 18 h), glycidamide (0.5 µM, 18 h), resveratrol (0.1 µM, 18 h), or a combination of acrylamide (1 µM) and resveratrol (0.1 µM, 18 h) or glycidamide (0.5 µM) and resveratrol (0.1 µM, 18 h). DNA damage was assessed by the comet assay modified by the addition of the FPG or hOGG1 enzyme. Significant levels of DNA damage were detected in the acrylamide treated spermatocytes in the presence of FPG (*F*_13,877_ = 92.5, ***p<0.001) or hOGG1 (**p<0.001). A significant increase in DNA damage was observed with glycidamide treatment in the presence of FPG (***p<0.001). Resveratrol treatment on its own had no effect on the level of DNA damage in spermatocytes with FPG or hOGG1 treatment. DNA damage assessed in cells treated with the combination of acrylamide and resveratrol was not significantly different from control. Treatment with glycidamide and resveratrol caused a significant induction in DNA damage when treated with FPG (***p<0.001), but not hOGG1. Spermatocytes treated with H_2_O_2_ (500 µM, 5 min) were used as a positive control for DNA damage. Significant increases in Tail DNA % were observed in cells treated with H_2_O_2_ in the presence of either FPG or hOGG1 (***p<0.001). All data are representative of n = 3 experiments (Mean ±SEM).

Resveratrol has been established as an inhibitor of CYP2E1 [37], [38]. The role of CYP2E1 in DNA adduct formation was assessed by the comet assay following treatment of isolated spermatocytes with acrylamide (1 µM), or glycidamide (0.5 µM) in the presence or absence of resveratrol (0.1 µM) for 18 h (Fig. 7). Use of the FPG enzyme in the comet assay revealed the formation of DNA adducts caused by acrylamide treatment (Fig. 5) and this was reduced by cotreatment with CYP2E1 inhibitor, resveratrol (Fig. 7). The addition of resveratrol to the glycidamide treatment had no effect on DNA adduct formation as detected by the FPG modified comet assay (Fig.7). Addition of the hOGG1 enzyme to the comet assay detected oxidative damage (Fig. 5). Resveratrol did not reduce the minimal oxidative effects caused by acrylamide treatment.

## Discussion

While steroidogenic enzymes such as CYP17A1 have previously been found in the germ line [39], our results are the first to demonstrate that detoxifying CYPs are also expressed at specific stages of meiotic male germ cell differentiation. Male germ cells were found to express both CYP2E1 mRNA and protein (Figs. 1 and 2). Previously, CYP2E1 protein was reported to be exclusively expressed in the interstitial cells of the testis [40]. However, in the present study, immunohistochemistry of mouse testis (Fig. 2 A) and isolated populations of germ cells (Fig. 2 B) clearly revealed protein expression of CYP2E1 in spermatocytes. The elevated expression of CYP2E1 in spermatocytes compared to other germ cells types may serve as increased cellular defence against chromosomal damage during this stage of germ cell development, as spermatocytes are entering into meiosis. However, while the presence of detoxifying enzymes in the male germ line may confer additional protection, it also comes with attendant risks. The generation of reactive metabolites as a product of detoxification processes may induce both cellular and DNA damage. In male germ cells, DNA damage is of particular importance as xenobiotic exposure may contribute to reduced fertility and impact on the health of future progeny [4].

The specificity of CYP2E1 for acrylamide enabled us to examine the role of one particular detoxifying enzyme in the male germ line in response to xenobiotic exposure. In the present study, we utilised a relatively low level of acrylamide exposure (1 µM, 18 h), that was not cytotoxic to isolated spermatocytes (Fig. 3), but sufficient to elicit a pronounced cellular response (Fig. 4). Spermatocytes were found to respond to acrylamide exposure by increasing mRNA expression not only of*CYP2E1*, but also*CYP1B1*. While CYP1B1 is not specifically involved in the metabolism of acrylamide, members of both the CYP1 and CYP2 families of CYPs are primarily involved in metabolism of xenobiotics and are typically induced as part of the toxic response [41]. Thus, it is possible that the toxic response triggered by acrylamide exposure increases expression of multiple genes involved in detoxification in spermatocytes. Indeed, regulation of various detoxifying CYPs has been observed in the liver of acrylamide exposed mice [42]. However, to our knowledge, the present study is the first to observe such a response in the male germ line.

The quantitative increase in CYP2E1 mRNA, observed in acrylamide treated spermatocytes suggested that CYP2E1 is upregulated in germ cells at the transcriptional level, through either transcriptional activation or via inhibition of mRNA degradation. Interestingly, several studies have demonstrated that the elevation of CYP2E1 protein levels by exogenous substrates in the liver is mediated largely through protein stabilisation, with little to no change in mRNA levels [43]. Conversely, other studies have indicated that induction of CYP2E1 gene expression does occur in extrahepatic tissues, such as kidney, intestine and lung [44], [45] which supports our observations in early male germ cells (Fig. 4). However, none of the aforementioned studies examined CYP2E1 induction in response to acrylamide, and induction of CYP2E1 in male germ cells has not previously been explored. The results of the current study indicate that male germ cells do respond to xenobiotic exposure and that specific mechanisms of CYP regulation are active in the mouse male germ line. Additionally, our results suggest that spermatocytes have the capacity to upregulate metabolism of acrylamide to glycidamide.

DNA damage in spermatocytes was investigated using the comet assay, which measures DNA strand breaks as opposed to adduct formation. Hence, cleavage enzymes that recognise specific DNA adducts were also utilised. The major DNA adducts that glycidamide forms are N7-(2-carbamoyl-2-hydroxyethyl)guanine (N7-GA-Gua) and N3-(2-carbamoyl-2-hydroxyethyl)adenine (N3-GA-Ade) [20]. The cleavage enzyme, FPG, has been used in previous studies in the comet assay to recognise these glycidamide adducts and introduce strand breaks at these adduct sites [46], [47]. In the absence of FPG, a significant increase in DNA damage was induced in spermatocytes treated with glycidamide (*p*<0.05, Fig. 5 B). This may be due to depurination of glycidamide adducts, resulting in single or double strand breaks which are detectable by the comet assay [48]. However, upon the addition of FPG, the level of DNA damage detected more than doubled for both acrylamide and glycidamide treated cells (Fig. 5 B) and was found to increase dose-dependently (Fig. 5 C and D). From this, it could be inferred that acrylamide induces DNA damage in spermatocytes via adducts, rather than DNA strand breaks, and may be indicative of the presence of glycidamide-DNA adducts as exposure to glycidamide in these cells produced similar increases in damage.

In contrast, a previous study by Hansen *et al*. [46] found that two hours of acrylamide exposure at millimolar concentrations did not induce DNA damage in dissociated testicular cells as detected by the FPG modified comet assay. These doses were 100 fold greater than the doses used in our study. However our study was conducted on a population of cells enriched for spermatocytes which express cyp2e1Thedifferences in exposure times between the two experiments may also be indicative of the required time for cells to metabolise acrylamide to glycidamide. As detailed above, germ cells respond by inducing greater levels of CYP2E1 mRNA. Therefore the longer exposure time in our experiments (18 h) may also lead to enhanced levels of CYP2E1, potentially upregulating the metabolic conversion of acrylamide to glycidamide. Indeed, Sega et al. [49] reported that a maximum peak in DNA repair activity in mouse testis was observed six hours post acrylamide treatment, which was considered to be related to the period of time needed for acrylamide metabolism to occur.

As previously mentioned, the damage detected by FPG in the comet assay could be indicative of oxidative adducts as well as glycidamide adducts as FPG has the capacity to recognise several types of DNA lesions, including 8-oxoGua. Exposure to either acrylamide or glycidamide may deplete glutathione levels and subsequently result in increased oxidative stress. Indeed, oxidative stress is known to contribute to poor sperm function and generate DNA damage in sperm [50]. However, cellular GSH levels were unaffected by either acrylamide or glycidamide in spermatocytes (Fig. 6 B). Further characterisation of oxidative DNA damage using hOGG1 failed to identify significant increases in the acrylamide treated spermatocytes (Fig. 6 A), and only a modest increase was found in glycidamide treated cells (22% Tail DNA, *p*<0.001). Hence, the DNA damage induced by acrylamide and glycidamide in spermatocytes are likely due to the presence of glycidamide adducts, and a minor contribution of oxidative damage.

Importantly, this study demonstrates the connection between CYP2E1 mediated metabolism of acrylamide and DNA damage. Resveratrol is an established inhibitor of CYP2E1 [37], [38]. While acrylamide treatment does cause an increase in DNA adducts, co-treatment of spermatocytes with 1 µM acrylamide and 0.1 µM resveratrol for 18 h reduced the level of DNA adducts as measured by the FPG modified comet assay to control levels. This reveals the capacity of isolated spermatocytes to metabolise acrylamide to glycidamide (Fig. 7). The levels of adduct formation when spermatocytes were treated with either glycidamide (0.5 µM) on its own or in combination with resveratrol were no different. Glycidamide is the molecule which forms adducts with DNA. Direct treatment with glycidamide causes DNA damage, with resveratrol treatment having no effect on the DNA damaging capacity of glycidamide only it's production by cyp2e1. Use of the hOGG1 enzyme in the comet assay demonstrated that resveratrol had no effect on the minimal role that oxidative adducts play in the DNA damaging capacity of acrylamide. Thus the isolated spermatocytes have the capacity to generate glycidamide adducts by metabolising acrylamide.

These findings are consistent with *in vivo* studies which suggest the relevance of acrylamide metabolism within the testis. Radioactively [^14^C] labelled acrylamide is detected in testis tissue only 1 hour after administration [11], showing that acrylamide reaches testicular tissue in its unmetabolised form. Thus, *in situ* activation of acrylamide to glycidamide in the i*n vivo* situation is highly likely. When we treat whole animals with acrylamide we see DNA damage in spermatocytes [51]. Since acrylamide can reach these cells without being metabolised our data indicates that localised metabolism is possible.

DNA damage is likely to be reversible in early stage germ cells, as DNA repair mechanisms are still intact [1]. However, post-meiotic cells may not have the capacity to repair DNA damage as effectively. The blood/testis barrier prevents the passage of most toxic substances through the seminiferous tubule towards the residing post-meiotic germ cells. Acrylamide however has been found to reach the testes in an un-metabolised state and is able to transit the blood/testis barrier, due to its low molecular weight and hydrophilicity [52]. Spermatogonia lie outside the blood/testis barrier (see Fig. S1 in File S1) and have functional repair mechanisms to deal with DNA damage. In contrast, spermatocytes, which lie within the blood/testis barrier, are in the process of undergoing meiosis and DNA damage generated at this stage would be expected to be of significant consequence. In a study by Olsen et al. [53], spermatocyte exposure to benzopyrene produced DNA damage that was retained throughout spermatogenesis, resulting in adducts present in the spermatozoa. Thus, it is highly possible that the DNA damage induced in spermatocytes by acrylamide will persist through to mature spermatozoa in mice exposed to acrylamide.

Induction of CYP gene expression in response to xenobiotic exposure in male germ cells represents an additional mechanism of cellular defence that has not been extensively examined in the germ line in previous studies. Having shown that spermatocytes are the specific germ cell type that express CYP2E1, it is interesting to note that in our previous study [23], chronic exposure to acrylamide in male mice also produced high levels of DNA damage in these cells. In the present study we also show that the presence of DNA adducts in spermatocytes following acrylamide exposure are likely due to glycidamide, rather than oxidative adducts. Indeed, the upregulation of *CYP2E1*expression in spermatocytes exposed to acrylamide may in turn upregulate the metabolism of acrylamide to glycidamide, leading to an increase in glycidamide-DNA adducts. This present study has demonstrated the capacity of spermatocytes to metabolise acrylamide to glycidamide via CYP2E1. This metabolism can be prevented with the use of the CYP2E1 inhibitor resveratrol. Importantly, the inhibition of glycidamide formation from acrylamide prevents the DNA damage as measured in terms of DNA adducts. These results provide further evidence of the consequences of acrylamide exposure during spermatogenesis and sheds light on the mechanisms of detoxification present in early male germ cells.

## Supporting Information

File S1 Five additional figures and a table. Table S1, Primers used for QPCR. Figure S1, Spermatogenesis. Figure S2, Cyp2e1 QPCR normalised to two reference genes. Figure S3, Optimisation of the Comet assay for FPG and hOGG1. Figure S4, Comet hedgehog frequency with FPG. Figure S5, Comet hedgehog frequency with hOGG1.
(DOCX)
