# Supplementary material for: Mouse Spermatocytes Express CYP2E1 and Respond to Acrylamide Exposure
Source: PLoS One. 2014 May 2;9(5):e94904. doi: 10.1371/journal.pone.0094904 (PMC4008485; doi:10.1371/journal.pone.0094904)
Supplement: File S1 — Five additional figures and a table. Table S1, Primers used for QPCR. Figure S1, Spermatogenesis. Figure S2, Cyp2e1 QPCR normalised to two reference genes. Figure S3, Optimisation of the Comet assay for FPG and hOGG1. Figure S4, Comet hedgehog frequency with FPG. Figure S5, Comet hedgehog frequency with hOGG1. (DOCX) [file pone.0094904.s001.docx]

Supplementary Data

**Table S1.** Primers used for QPCR including their product length and annealing temperatures for *cyclophilin*, *hypoxanthine guanine phosphoribosyl transferase (Hprt)*, *cytochrome P450 Cyp2e1* and *cytochrome P450 Cyp1b1*.

| Gene | Forward Primer | Reverse Primer | Product Length (bp) | Annealing Temp. (°C) | Accession No. |
| --- | --- | --- | --- | --- | --- |
| *Cyclophilin* | CGTCTCCTTCGAGCTGTTT | ACCCTGGCACATGAATCCT | 100 | 50-60 | NM_008907.1 |
| *Hprt* | GTCATGAAGGAGATGGGAG | ATCTACAGTCATAGGAATGG | 137 | 50-60 | NM_0.13556.2 |
| *Cyp2e1* | CGCATGGAACTGTTTCTGC | CAATTGTAACAGGGCTGAGGTC | 100 | 59 | NM_021282.2 |
| *Cyp1b1* | GTCTGTGAATCATGACCCAGC | ACAGTTCCTCACCGATGCAC | 152 | 59.4 | NM_009994.1 |
|  |  |  |  |  |  |

Supplementary Data

**A.**


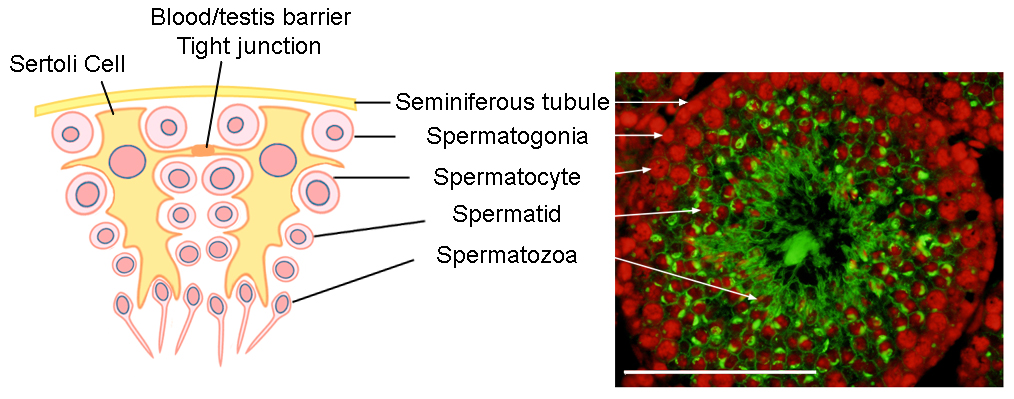


**B.**


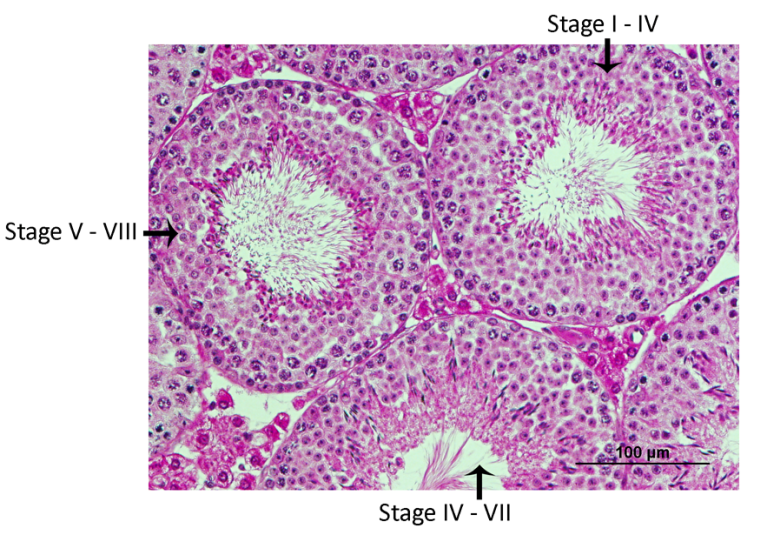


**Fig. S1.** **A)** Diagram outlining where male germ cells reside in the seminiferous tubule at different stages of spermatogenesis in the mouse. Also depicted are the supporting Sertoli cells and the tight junctions between Sertoli cells, which form the blood/testis barrier. Section of untreated mouse testis (shown on right) was stained with FITC-PNA, which labels the developing acrosome (green) and counter-stained with PI (red) to distinguish between different male germ cell types in the testis. **B)** Tubule staging in a mouse testis section stained with hematoxylin and eosin stain as identified according to the stages for the production of spermatozoa in the mouse seminiferous epithelium (Russel et al., 1990). Based on visual inspection, the tubule presented in (**A)** is in stage V – VIII, indicating that from the basement membrane to the lumen, germ cells can be identified as type B spermatogonia, pachytene spermatocytes, step 5 to 6 round spermatids and step 15 elongating spermatids.

Supplementary Data


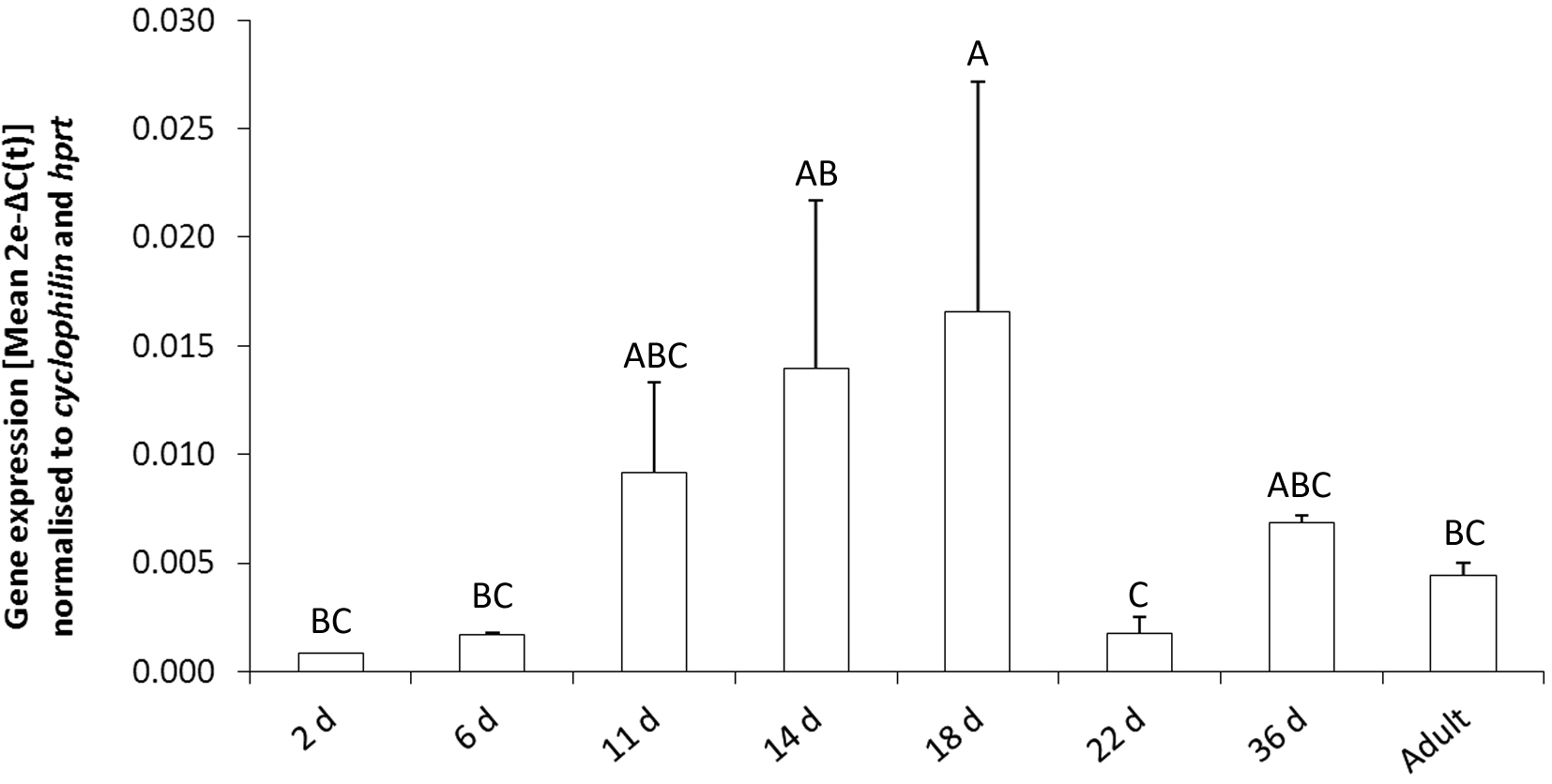


**Fig. S2:** Q-PCR analysis of *Cyp2e1* mRNA expression normalised to the geometric mean of reference genes cyclophilin and hprt in mouse testis at different developmental stages 2, 6, 11, 14, 18, 22, and 36 d after birth, and adult (older than 56 d). Normalisation to two reference genes produced a similar *Cyp2e1* expression profile in developing testis, validating the data presented in Fig. 1. Data are representative of n = 2 experiments and depicted as transformed values, 2e-∆C(t) (Mean ±SEM). Capital letters indicate statistically significant differences between groups (*F*_7,34_ = 4.4, *p* < 0.05).

Supplementary Data

**
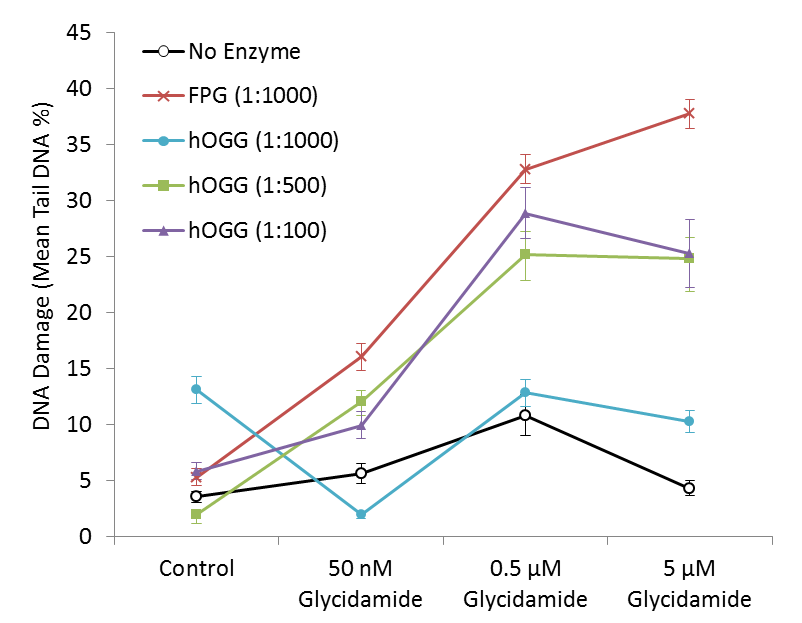
**

FPG concentration used in study

hOGG1 concentration used in study

**A.**

**B.**

hOGG1 concentration used in study

FPG concentration used in study

**Fig. S3:** Optimisation of FPG and hOGG1 in the Comet assay, conducted on spermatocytes treated with glycidamide at 50nM, 0.5µM, 5µM for 18 h, or H_2_O_2_ at 50nM, 500nM, 5µM for 5 min. **(A)** Enzyme activity of FPG was confirmed as the addition of FPG increased detection of DNA damage in glycidamide treated cells. Enzyme activity of hOGG1 could be confirmed as increases in DNA damage were detected following hOGG1 treatment at 1:100 and 1:500, but not at 1:1000 dilution in glycidamide treated cells. **(B)** Both FPG and hOGG1 detected similar levels of damage in H_2_O_2_ treated cells at the concentrations of used in our study (1:1000 and: 500 respectively). However increased hOGG concentration (1:100) detected more damage than FPG, demonstrating not only the activity of the hOGG enzyme but also the specificity of hOGG1 for oxidative damage.

Supplementary Data


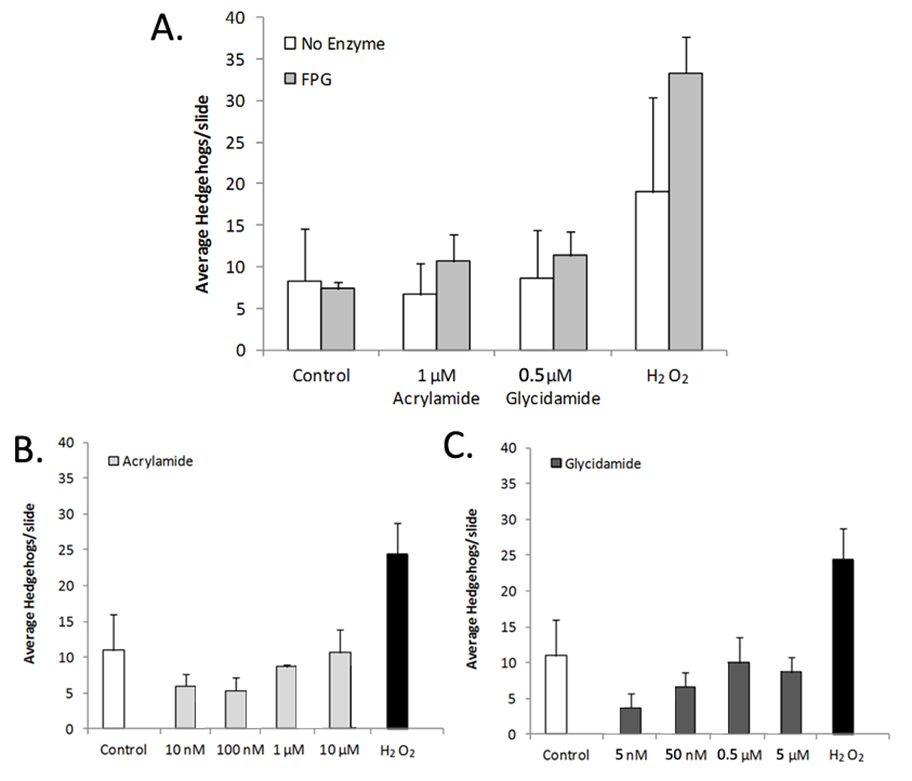


**Fig. S4.** **(A)** Frequency of ‘hedgehog’ comets observed in the Comet assay ± FPG enzyme (Fig. 5 B). **(B)** Frequency of ‘hedgehog’ comets observed in the Comet assay + FPG enzyme, with increasing concentrations of acrylamide or **(C)** glycidamide (Fig. 5 C, D). The hydrogen peroxide positive control treatment for DNA damage had the highest frequency of ‘hedgehog’ comets; however, no statistically significant differences were found across all samples.

Supplementary Data


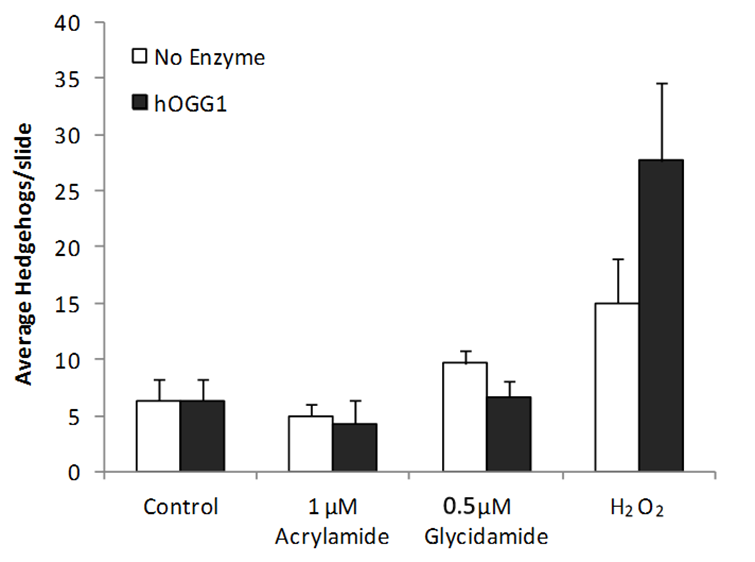


**Fig. S5.** Frequency of ‘hedgehog’ comets observed in the Comet assay ± hOGG1 enzyme (Fig. 6A). The hydrogen peroxide positive control treatment for DNA damage had the highest frequency of ‘hedgehog’ comets; however, no statistically significant differences were found across all samples.
